# Dedicated venous stent placement across the thoracic outlet: will novel venous stents transform axillosubclavian vein thrombosis treatment paradigm?

**DOI:** 10.1186/s42155-025-00577-4

**Published:** 2025-08-05

**Authors:** Gabriel E. Li, Mayura P. Umapathy, David S. Shin, Matthew Abad-Santos, Eric J. Monroe, Jeffrey Forris Beecham Chick, Mina S. Makary

**Affiliations:** 1 Section of Vascular and Interventional Radiology, Department of Radiology, University of Washington, 1959 Northeast Pacific Street, Seattle, WA 98195 USA; 2 Division of Vascular and Interventional Radiology, Department of Radiology, The Ohio State University Wexner Medical Center, Columbus, OH 43210 USA; 3 Division of Vascular and Interventional Radiology, Department of Radiology, University of Southern California, 1500 San Pablo Street, Los Angeles, CA 90033 USA; 4 Department of Radiology, University of Wisconsin, 1675 Highland Ave, Madison, WI 53792 USA

**Keywords:** Axillosubclavian vein thrombosis, Endovascular stenting, Dedicated venous stents, Venous obstruction, Thoracic outlet syndrome, Thoracic outlet, Thoracic central venous obstruction

## Abstract

**Background:**

Endovascular stenting is widely accepted as the standard treatment for central venous obstruction syndromes such as superior vena cava (SVC) syndrome due to its demonstrated clinical efficacy and improved patient outcomes. However, its application in axillosubclavian vein thrombosis (ASVT) has been limited due to concerns about stent compression within the thoracic outlet. This report aims to evaluate the feasibility and safety of the off-label use of dedicated venous stents—engineered with enhanced mechanical features—as an alternative endovascular approach for the treatment of ASVT.

**Methods:**

Thirty-eight patients (43 affected limbs) with symptomatic ASVT and no prior treatment or surgical decompression underwent endovascular placement of dedicated venous stents across the thoracic outlet with Abre Venous Stents (Medtronic, Dublin, Ireland), Venovo Venous Stents (BD, Franklin Lakes, NJ, USA), or Vici Venous Stents (Boston Scientific, Marlborough, MA, USA). Stents were extended peripherally to the subclavian or axillary veins and centrally to the brachiocephalic vein or SVC. Technical success was defined as successful stent deployment across the costoclavicular space, and clinical success as symptomatic improvement.

**Results:**

Stent placement was technically successful in all 43 limbs (100%), with clinical improvement observed in 97.4% of patients. The one patient without clinical improvement experienced early thrombosis of the stent, requiring mechanical thrombectomy and additional stenting. Follow-up CT venography at a mean of 301.3 days demonstrated high primary stent patency rates (81.4%), with stent crushing observed in only 7.0% of limbs and no instances of stent fracture. Adverse events were limited, including two access site hematomas and one hypotensive episode, all of which resolved without evidence of long-term complications.

**Conclusions:**

Our findings suggest that stenting across the thoracic outlet for the treatment of ASVT may be a viable option with the use of novel dedicated venous stents, potentially expanding treatment strategies for TCVO.

## Background

Endovascular stenting has become the standard of care in various central venous obstruction syndromes such as superior vena cava (SVC) occlusive disease due to its minimally invasive approach and tendency to elicit rapid symptom relief [1]. In contrast, this approach has been limited in axillosubclavian vein thrombosis (ASVT), a form of thoracic central venous obstruction (TCVO), due to concerns about stent compression by adjacent musculoskeletal structures in the thoracic outlet, risking stent fracture, deformation, malposition, or occlusion [2]. However, recent success of upper extremity and central venous recanalization with dedicated venous stents—engineered to balance flexibility, radial force, and crush resistance—presents an opportunity to reevaluate this application in ASVT [1, 3, 4].

This report describes the use of dedicated venous stents during recanalization for ASVT with the aim of evaluating the feasibility, safety, and potential for sustained patency of this novel approach to endovascular stenting across the thoracic outlet.

## Methods

This retrospective study included 38 patients (43 affected limbs) who underwent endovascular stenting with dedicated venous stents across the thoracic outlet for ASVT, which was diagnosed with evaluation of clinical manifestations and imaging studies. Presenting symptoms included facial or upper extremity swelling (76.3%), upper extremity pain (21.1%), dyspnea (15.8%), and hemorrhage (5.3%). Thirteen patients (34.2%) also exhibited symptoms consistent with SVC syndrome. Pre-procedural evaluation included contrast-enhanced CT and duplex venous ultrasound to characterize the anatomy and degree of venous obstruction. None of the patients underwent previous treatment for ASVT or surgical resection of the first rib prior to stenting.

Procedures were performed under general anesthesia, and access was obtained via right femoral vein under ultrasound guidance. Brachial or jugular access approaches were additionally utilized in complex cases to enhance procedural control. A 10 F vascular sheath was then placed, and central diagnostic venography was performed. Next, guidewires and directional catheters were used to recanalize the occlusive lesion, followed by intravascular ultrasound (IVUS) evaluation to understand the central occlusion and landing zones within the costoclavicular space, which was used in addition to fluoroscopic landmarks for the thoracic outlet. This step was followed by balloon angioplasty to predilate the stenotic segment, with 10 to 16 mm balloons.

**Fig. 1:**
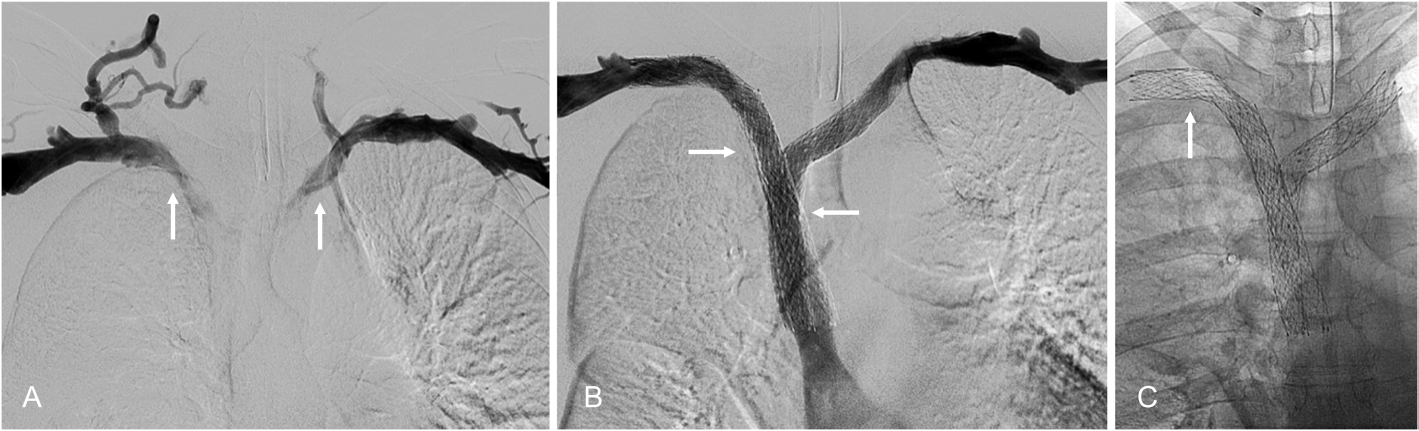
*64-year-old male with cirrhosis post transjugular intrahepatic portosystemic shunt placement with superior vena cava syndrome* (**A**) Bilateral upper extremity venography demonstrating bilateral brachiocephalocaval vein occlusion (arrows) (**B**) Bilateral upper extremity venography, after bilateral brachiocephalocaval stent reconstruction using bilateral 12-mm and 14-mm Abre Venous Stents (arrows), showing brisk in-line from both upper extremities to the right atrium (**C**) Radiograph demonstrating the 14-mm Abre stent in the thoracic outlet (arrow) without deformation or fracture

**Fig. 2:**
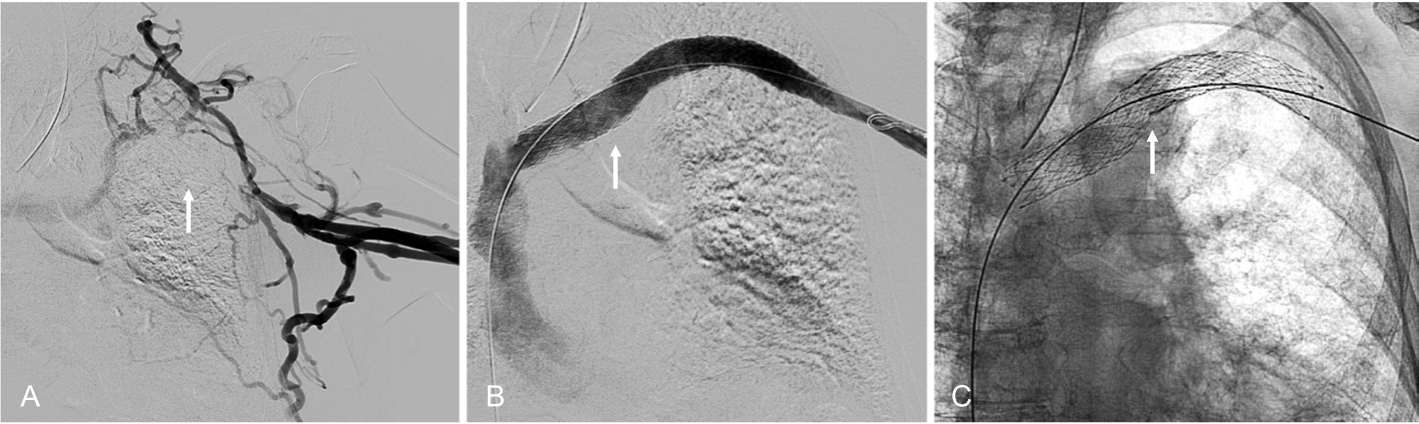
*76-year-old man with recurrent left axillosubclavian venous thrombosis and left upper extremity swellin*g (**A**) Left upper extremity venography demonstrating left axillosubclavian venous occlusion (arrow) (**B**) Left upper extremity venography, after left axillosubclavian stent reconstruction using 12-mm and 14-mm Abre Venous Stents (arrow), showing brisk in-line from the left upper extremities to the right atrium (**C**) Radiograph demonstrating the 12-mm and 14-mm Abre stent in the thoracic outlet (arrow) without deformation or fracture

**Fig. 3:**
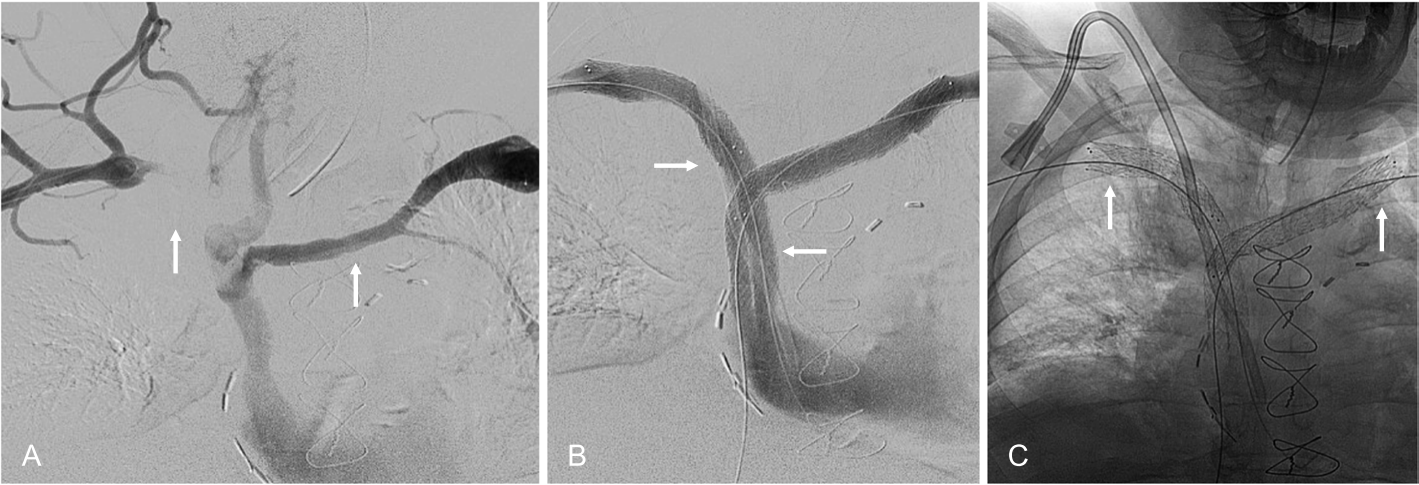
*65-year-old male with end-stage renal disease with superior vena cava syndrome* (**A**) Bilateral upper extremity venography demonstrating bilateral brachiocephalocaval vein occlusion (arrows) (**B**) Bilateral upper extremity venography, after bilateral brachiocephalocaval stent reconstruction using bilateral 11-mm VBX balloon expandable endoprostheses (centrally) and 14-mm Abre Venous Stents (peripherally) (arrows), showing brisk in-line from both upper extremities to the right atrium (**C**) Radiograph demonstrating the 14-mm Abre stents in the thoracic outlets (arrow) without deformation or fracture

**Fig. 4:**
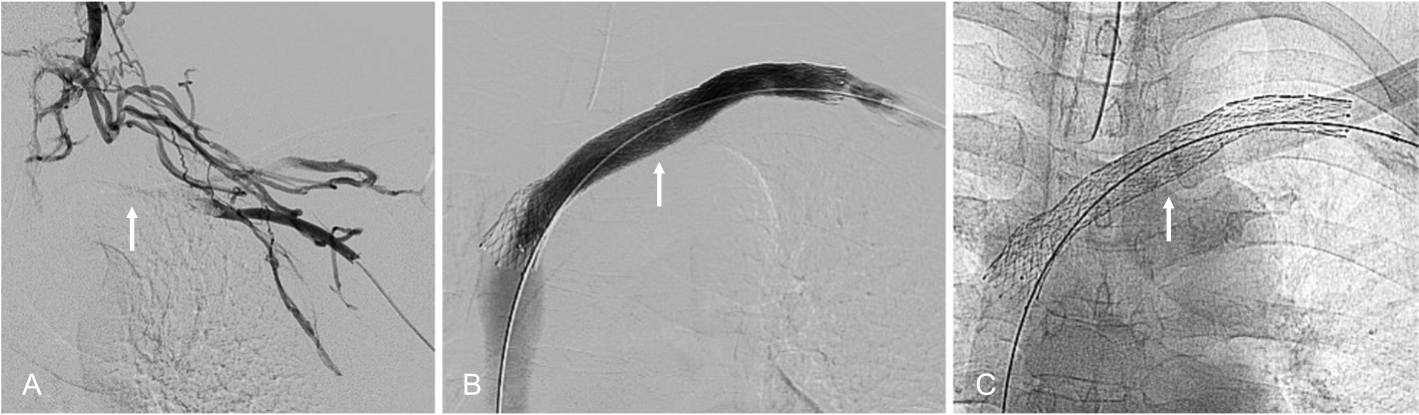
*50-year-old man with recurrent left axillosubclavian venous thrombosis and left upper extremity pain and swellin*g (**A**) Left upper extremity venography demonstrating left axillosubclavian venous occlusion (arrow) (**B**) Left upper extremity venography, after left axillosubclavian stent reconstruction using 14-mm Venovo Venous Stents (arrow), showing brisk in-line from the left upper extremities to the right atrium (**C**) Radiograph demonstrating the 14-mm Venovo stent in the thoracic outlet (arrow) without deformation or fracture

Dedicated venous stents, self-expandable stents initially designed for use in iliofemoral veins, were subsequently deployed off-label in this case series based on anatomical and clinical requirements. A total of 53 dedicated venous stents were placed, including 29 (54.7%) Abre Venous Stents (Medtronic, Dublin, Ireland), (Figs. 1, 2, and 3), 20 (37.7%) Venovo Venous Stents (BD, Franklin Lakes, NJ, USA) (Fig. 4), and four (7.5%) Vici Venous Stents (Boston Scientific, Marlborough, MA, USA). The mean stent diameter was 13.4 ± 1.3-mm (range: 12–16-mm), and the mean stent length was 80.5 ± 23.9-mm (range: 60–150-mm). Implanted stent constructs were extended peripherally to the subclavian vein in 21 limbs (48.8%) and the axillary vein in 22 limbs (51.2%), as well as centrally to the brachiocephalic vein in 11 limbs (25.6%) and the SVC in 32 limbs (74.4%). After stent positioning, an additional angioplasty was conducted for full stent deployment.

Diagnostic venography and IVUS were repeated to confirm proper apposition to the vessel wall and check for residual stenosis. Stent expansion and luminal conformity were also confirmed. Following successful post-deployment assessment, all access sites were removed. Hemostasis was achieved using manual pressure. Patients were not heparinized during the procedure. Post-procedurally, all patients were managed on aspirin therapy, with 37 patients (97.4%) receiving 81 mg daily and one patient (2.6%) receiving 325 mg daily. Ten patients (26.3%) were managed with aspirin monotherapy. A subset of patients received additional therapy based on clinical presentation, including clopidogrel in 5 patients (13.2%) and anticoagulation in 26 patients (68.4%). Among the patients receiving anticoagulation, 14 were treated with a direct oral anticoagulant, 11 with enoxaparin, and one with warfarin. Clopidogrel was continued for two months, while aspirin was prescribed as lifelong therapy. The duration of anticoagulation therapy varied based on individual patient risk factors and clinical history. All patients underwent follow-up imaging with chest CT venography (mean interval: 301.3 ± 351.5 days; range: 4–1548 days) to confirm stent patency and assess for stent crushing or fracture.

Technical success was defined as successful deployment of the stent across the costoclavicular space, as a fluoroscopic landmark for the thoracic outlet. Clinical success was defined as symptomatic improvement, assessed based on resolution or reduction of initial presenting symptoms at the first clinic follow-up, typically one month post-intervention. Adverse events were recorded and classified according to the Society of Interventional Radiology (SIR) Adverse Event Classification System, 2017 Modification [5].

## Results

Thirty-eight patients with 43 affected limbs underwent stenting across the thoracic outlet with dedicated venous stents. Stent diameters ranged from 12–16 mm (mean 13.4 ± 1.3 mm) and lengths from 60–150 mm (mean 80.5 ± 23.9 mm). In cases requiring overlapping stents, a 3 cm overlap was used. One patient received three overlapping stents in both limbs, while other cases involved two stents. Stents protruded 6–10 mm into the costoclavicular space.

Technical success was achieved in 43 limbs (100%). Clinical success was achieved in 37 patients (97.4%). In the single (2.6%) patient without clinical improvement, post-procedural imaging demonstrated early thrombosis of the stent construct, which required mechanical thrombectomy followed by additional stent placement using two 8-mm Viabahn Endoprosthesis stent-grafts (Gore Medical, Newark, DE, USA) and a 12-mm Abre stent. Immediate adverse events occurred in three patients (7.9%), including two minor, self-limited access site hematomas (SIR Grade 1) and one case of hypotension requiring norepinephrine support (SIR Grade 2).

During chest CT venography at a mean follow-up of 301.3 ± 351.5 days (range: 4–1548 days), primary and primary-assisted stent patency were each 81.4% (35/43 limbs), while secondary patency was 100%. Stent crushing occurred in three limbs (7.0%) in three different patients, with occlusion in one of these cases. No stent fractures were observed. Zero stent migrations were noted. No definitive correlations were found between stent crushing and either stent overlap length or degree of protrusion into the costoclavicular space.

Eight limbs (18.6%) required reintervention due to occlusion at a mean of 214.9 ± 351.4 days post-intervention (range 6–1071 days). These procedures included venoplasty in eight limbs (100%), additional stenting in six limbs (75%) using nine dedicated venous stents of the same type as originally implanted, and thrombectomy in four limbs (50%) using Arrow-Trerotola, Indigo Lightning CAT (Penumbra Inc., Alameda, CA, USA) and Flowtriever (Inari, Cambridge, MA, USA) systems. One patient also underwent catheter-directed thrombolysis with 10 mg tissue plasminogen activator. No patients required subsequent surgical decompression. No significant differences in outcomes were observed based on the type of dedicated venous stent used.

## Discussion

ASVT is a form of TCVO that may result in acute upper extremity pain and swelling, pulmonary embolism, or post-thrombotic syndrome [6]. The mainstay treatment is anticoagulation, with possible additional therapy, such as thrombectomy, thrombolysis, or surgical decompression. Endovascular stenting in ASVT is typically reserved for select cases [2, 7]. Often, it is used for recurrent thrombosis in poor surgical candidates and follows prior first rib resection, as stenting across the thoracic outlet has historically been contraindicated due to risks of stent crushing or fracture from anatomical compression [2]. This limitation is significant given that stenting has been associated with faster recovery and symptom relief in other central venous occlusive diseases, such as SVC syndrome. However, the development of dedicated venous stents—designed with enhanced mechanical properties—may improve the feasibility of stenting in anatomically complex regions like the thoracic outlet.

This retrospective study evaluates the off-label use of dedicated venous stents, originally designed for iliofemoral venous disease, across the costoclavicular space in patients with symptomatic ASVT without prior surgical decompression or treatment. Our results suggest that stenting across the thoracic outlet with these stents may be technically feasible and safe in select patients, with no major adverse events observed. High rates of technical success (100%), clinical success (97.4%), and stent patency (primary/primary-assisted: 81.4%; secondary: 100%) without stent fractures over a mean imaging follow-up of 301.3 days support their potential use in this context.

It is important to note that follow-up was relatively short, and further research with longer-term data is needed to assess the clinical durability of this approach and compare outcomes with existing literature. Prior studies on venous stenting in venous thoracic outlet syndrome (vTOS) reported primary stent patency rates around 66% at three years, though those cohorts included patients who underwent surgical decompression [8]. Our results during the current follow-up period compare favorably with existing literature. In a 2022 series by de Boer et al., primary patency at 10 months was approximately 87–88% [8]. Notably, their cohort underwent first rib resection, and some patients (6.1%) received non-dedicated venous stents. In contrast, our cohort received only dedicated venous stents without surgical decompression, yet demonstrated a comparable primary patency (81.4%) at a similar time point.

This comparison raises the question of how outcomes differ between the traditional strategy of decompression followed by venous stenting, and the less invasive approach presented here for ASVT. Further research comparing these two strategies is needed to clarify the best long-term approach. Comparative analyses between stenting, anticoagulation, and decompression for ASVT are also needed to refine patient selection criteria for this novel strategy. Additionally, further investigation is warranted to better understand potential associations between dedicated venous stent deformation in this application and specific procedural factors, including stent overlap length and the degree of stent protrusion into the costoclavicular space.

## Conclusions

Although larger studies with longer follow-up are needed, our findings suggest that—with the off-label use of dedicated venous stents—stenting across the costoclavicular space may no longer be an absolute contraindication when necessary to ensure adequate inflow during TCVO reconstruction.

## Data Availability

The datasets used and/or analyzed during the current study are available from the corresponding author on reasonable request.

## Abbreviations

SVC: Superior Vena Cava
ASVT: Axillosubclavian vein thrombosis
TCVO: Thoracic central venous obstruction
IVUS: Intravascular ultrasound
SIR: Society of Interventional Radiology
vTOS: Venous thoracic outlet syndrome
